# 1087. Imipenem-Cilastatin-Relebactam (I/R) Pharmacokinetics (PK) in Critically Ill Patients with Augmented Renal Clearance (ARC)

**DOI:** 10.1093/ofid/ofab466.1281

**Published:** 2021-12-04

**Authors:** Andrew J Fratoni, John W Mah, David P Nicolau, Joseph L Kuti

**Affiliations:** 1 Hartford Hospital, Hartford, Connecticut; 2 Center for Anti-Infective Research and Development, Hartford Hospital, Hartford, Connecticut

## Abstract

**Background:**

Imipenem (IMI) and relebactam (REL) are predominantly excreted via glomerular filtration. ARC is a common syndrome in critically ill patients with sepsis, whereby increased renal blood flow may result in enhanced solute clearance; therefore, sub-therapeutic antibiotic concentrations are of concern. Herein, we describe the PK of I/R in critically-ill patients with confirmed ARC.

**Methods:**

Infected patients in the intensive care unit with ARC (CrCl ≥130 mL/min) received a single dose of I/R 1.25g as a 30min infusion. Blood samples were collected over 6 hours (hr) for IMI and REL concentration determination by a validated LC/MS/MS assay. Protein binding was assessed at 0.5hr by ultrafiltration (UF). An 8hr urine creatinine (UCr) collection was performed to confirm ARC. IMI and REL plasma concentrations were fitted to compartmental models in WinNonlin. Simulated concentration vs time profiles were used to assess attainment of pharmacodynamic (PD) targets for IMI (30%*f*T >MIC) and REL (*f*AUC:MIC 18) at the susceptibility breakpoint of 2 mg/L.

**Results:**

Five patients (60% female) completed the study. Mean (SD) age, weight, and APACHE II were 43 (14) years, 90 (15) kg, and 16 (6), respectively. All patients had confirmed ARC with CrCl of 160.6 ± 47.0 mL/min (range: 135-244mL/min) based on UCr. Both IMI and REL concentrations fitted a 2-compartment better than 1-compartment model. IMI PK was: clearance, 17.9 ± 8.7 L/hr; volume of central compartment, 15.6 ± 11.2 L; volume of peripheral compartment, 10.6 ± 5.4 L; and intercompartmental clearance, 16.6 ± 14.5 L/hr. REL PK parameters were 11.9 ± 7.5 L/hr, 17.0 ± 11.3 L, 13.5 ± 9.9 L, and 13.4 ± 11.1 L/hr, respectively. Half-life was 1.5 ± 0.5 for IMI and 2.8 ± 2.2 hr for REL. Protein binding for IMI ranged from 0-10%, while REL was 0-14%. IMI *f*T >MIC ranged from 40-90%, and REL *f*AUC:MIC ranged from 22.6-59.0.

**Conclusion:**

These are the first data to describe IMI and REL PK in critically-ill infected patients with ARC. Despite plasma clearance values greater than those reported in healthy volunteers and patients in clinical trials, I/R 1.25g as a 30 minute infusion provided optimal exposure in all patients for isolates with MICs ≤2 mg/L.

**Disclosures:**

**David P. Nicolau, PharmD**, **Abbvie, Cepheid, Merck, Paratek, Pfizer, Wockhardt, Shionogi, Tetraphase** (Other Financial or Material Support, I have been a consultant, speakers bureau member, or have received research funding from the above listed companies.) **Joseph L. Kuti, PharmD**, **Allergan** (Speaker’s Bureau)**BioMérieux** (Consultant, Research Grant or Support, Speaker’s Bureau)**Contrafect** (Scientific Research Study Investigator)**GSK** (Consultant)**Merck** (Research Grant or Support)**Paratek** (Speaker’s Bureau)**Roche Diagnostics** (Research Grant or Support)**Shionogi** (Research Grant or Support)**Summit** (Scientific Research Study Investigator)

